# Effects of Ilicis Chinensis folium extract supplementation on growth performance, serum parameters, intestinal morphology, and antioxidant capacity of broiler chickens

**DOI:** 10.1186/s12917-023-03667-4

**Published:** 2023-07-26

**Authors:** Yingjie Zhong, Liang Li, Wujun Chen, Dongming Xing, Xiaolin Wu

**Affiliations:** 1 Department of Orthopedics, Cancer Institute, The Affiliated Hospital of Qingdao University, Qingdao University, Qingdao, China; 2 Foss Analytical Co.Ltd, Beijing, China; 3 School of Life Sciences, Tsinghua University, Beijing, China; 4 Department of Orthopedics, The Affiliated Hospital of Qingdao University, Qingdao, China

**Keywords:** ICFE, Growth performance, Intestinal morphology, Broiler chickens

## Abstract

**Background:**

*Ilicis chinensis* folium extract (ICFE) is a powder extracted and processed with *Ilex chinensis Sims* (ICS) which has numerous bioactivities and is conventionally used in traditional Chinese medicine. Nonetheless, there has been no definitive study evaluating ICFE’s application as a feed supplement for broilers. This research sought to determine the chemical composition and evaluate how dietary ICFE supplementation affects the growth performance, serum metrics, intestinal structure, and antioxidant capacity of broilers.

**Methods:**

A total of 360 one-day-old broiler chicks were assigned to four treatments (with 9 replicates of 10 chicks, each) of dietary supplementation with ICFE at 0, 250, 500, and 1,000 mg /kg for 42 days.

**Results:**

Ten polyphenolic compounds and two triterpenoid glycosides were detected by HPLC. In the grower stage and overall, broilers supplemented with 500 and 1,000 mg/kg ICFE exhibited a higher ADFI (*P* < 0.05) than the controls. Additionally, compared to the controls, broilers receiving low, medium, or high dosages of ICFE exhibited higher average daily gains (*P* < 0.05) throughout the starter stage and overall. Organ indices showed no significant variation, suggesting that ICFE was non-toxic. ICFE supplementation increased the height of villi in the duodenum and jejunum, reduced crypt depth, and increased the villus/crypt ratio in the duodenum (*P* < 0.05). Serum concentrations of IL-4 and IgA were increased in ICFE-supplemented broilers. The serum malondialdehyde concentration was reduced, whereas superoxide dismutase activity and total antioxidant capacity increased through supplementation with ICFE.

**Conclusion:**

ICFE supplementation can improve intestinal morphology, antioxidant capacity, and growth performance of broilers. Hence, ICFE is a promising and safe alternative to antibiotics in broilers, and 500 mg/kg appears to be the optimal dose.

## Background

Abuse of feed antibiotics has given rise to bacterial resistance, which poses a threat to human health. With the increasing demand for better meat quality and physical health, many countries have imposed a ban on antibiotic growth promoters (AGPs). Natural alternatives to antibiotics including probiotics, herbs, botanicals, organic acids, essential oils, and enzymes are the most common feed additives that are increasingly used in poultry industry, apart from AGPs.

Traditional Chinese medicine (TCM), which relies on medicinal herbs, has long been recognized as an essential source in promoting and sustaining health in humans and animals [1–4]. Plant extracts used in Chinese medicine can improve several aspects of livestock, including growth, appetite, immunity, and antioxidative characteristics [5, 6]. Thus, Chinese medicine extracts may be of use as functional feed supplements in the poultry sector. Such extracts are typically considered risk-free, efficient, and easily available, which makes them promising candidates for use in antibiotic-replacement research and practice.

*Ilex chinensis Sims* (ICS) belongs to the *Aquifoliaceae* family and is used to treat lung heat, cough, sore throat, heat strangury, diarrhea, and empyrosis. Previous studies on ICS have revealed several particular and structurally diverse metabolites, such as triterpenoids, phenolic acids, flavonoids, cyanogenic glucosides, and hemiterpene glucosides [7, 8]. The pharmacological effects of ICS include antibacterial, antioxidant, hepatoprotective, and hypolipidemic activity [9, 10]. Many TCM extracts have been recognized as beneficial feed supplements in recent years due to their capacity of enhancing animal productivity and increasing the quality of poultry products [11–13]. *Ilicis chinensis* folium extract (ICFE) is a powdered extract from ICS. However, studies on the effects of ICS and ICFE on animal production are lacking. We hypothesized that enhancing broiler diets with ICFE would lead to better growth performance through enhanced antioxidant capacity and improved intestinal morphology. We thus assessed the content of active ingredients in ICFE and examined how ICFE supplement influenced intestinal morphology, growth performance, and antioxidant capacity in broiler chickens.

## Methods

### Analysis of the chemical composition of ICFE

A powdered form of ICFE synthesized in the laboratory was included in the diets of broilers. High-performance liquid chromatography (HPLC) was used to r determine the chemical composition. Detection and quantification were achieved using an LC-20AD system controller (Shimadzu, Japan), a CTO-20 A column heater, a DGU-20A5R degasser, a LC-20AT pump, a SIL-20 A autosampler, and diode array detector set at 254 nm. Agilent ZORBAX SB-C_18_ column (250*4.60 mm, 5 μm) was used and the column temperature was adjusted to 30 °C, the flow rate was 1.0 mL/min, and the injection volume was 10 µL. Acetonitrile (A) and acetic acid dissolved in water at a concentration of 0.1% (B) were the mobile phase. The following conditions were used for the gradient program: 95% (B) in 0–20 min, 95–60% (B) in 20–50 min, 60% (B) in 50–60 min, 60–95% (B) in 60–61 min, 95% (B) in 61–65 min. The data were analyzed using the Shimadzu LabSolutions Automated Software System.

### Experimental design, animals, and diets

A total of 360 Lohmann commercial broilers aged one day were procured from a commercial chick producer (New Hope Group, Linyi, China) and individually weighed and randomly allotted to one of four treatments for a 42-day feeding study.

Nine replicas (i.e., cages) were used per treatment, and each cage contained 10 broilers. Mashed feed and water were available to the broilers at all times. The broilers were kept in a climate-controlled facility with an 18/ h light/dark cycle and relative humidity of 45–65%. The temperature during the first seven days of the experiment was 34 °C and 25 °C for the remaining time. Vaccinations were administered according to routine immunization procedures; one-day-old broilers were injected with inactivated Newcastle disease vaccine and bursa Fabricius vaccine, and 14-day-old broilers were injected with avian influenza vaccine. ICFE was added to a corn-soybean meal base diet for broilers at the following levels: 0 (control group), 250, 500, and 1,000 mg/kg, correspondingly. The diet of each group was prepared to contain the nutrients as per the recommendation of the NRC (1994). The ingredients and nutrient content of the corn-soybean meal-based basal diet (the starter and the grower diets) are shown in Table 1. On days 21 and 42, after fasting for 12 h, we weighed all broilers in each cage. To determine the average daily feed intake (ADFI), the average daily gain (ADG), and the feed conversion rate (FCR), the feed consumption was assessed per cage during the starter (0–21 days), grower (22–42 days), and overall (0–42 days) periods.

**Table 1:** Composition and nutrient level of basal diet (as-fed basis)

Items	Starter (1 to 21 d)	Grower (22 to 42 d)
Ingredients, %		
Corn	58.41	63.92
Soybean meal(43%CP)	33.78	28.05
Soybean oil	2.90	3.00
Fish meal (60.2%CP)	1.51	1.51
Dicalcium phosphate	1.40	1.40
Sodium chloride	0.21	0.21
DL-Methionine	0.19	0.19
Limestone	1.10	1.22
premix^1^	0.50	0.50
Total	100	100
Nutrient composition		
ME^2^(kcal/kg)	3050	3106
Crude protein (%)	21.05	19.17
Calcium (%)	0.96	0.94
Total phosphorus (%)	0.66	0.64
Lys	1.18	0.97
Met	0.51	0.45

^1^The premix provided the following (per kilogram of compound feed): vitamin A, 8,050 IU; cholecalciferol, 1,800 IU; vitamin E, 20 IU; vitamin K3, 5.10 mg; thiamine, 2.40 mg; riboflavin, 8.20 mg; pantothenic acid, 15.30 mg; pyridoxine, 3.10 mg; cobalamin, 0.02 mg; niacin, 32 mg; choline chloride, 1,000 mg; biotin, 0.20 mg; folic acid, 1.20 mg; Mn, 68 mg; Fe, 85 mg; Zn, 58 mg; Cu, 8.60 mg; I, 0.27 mg and Se, 0.20 mg

^2^ME based on calculated values; others were analyzed values

### Collection and measurements of samples

After fasting for 12 h on days 21 and 42 of the trial, a random selection of 18 chicks (2 per cage) was done from each group in the morning. Each bird’s wing vein was punctured to collect 5 mL blood which was placed in an anticoagulant-free Vacutainer tube. Serum was separated through 15 min of centrifugation at 4000 rpm after being incubation at 37 ℃ for 2 h and the serum samples were then stored at -80 ℃ till subsequent usage.

After blood sampling, the birds were anesthetized through intravenous injection with sodium pentobarbital (30 mg/kg; Sigma-Aldrich, St. Louis, MO, USA) [14] and were then killed through decapitation. The abdominal cavities were opened immediately, and the hearts, livers, and kidneys were retrieved for organ index assessment. The entire small intestine was then immediately excised and was separated from the surrounding connective and mesentery tissues. For histological analysis, we retrieved approximately 2-cm-long samples from the mid-sections of each broiler’s ileum, jejunum, and duodenum and preserved them in 4% paraformaldehyde buffer.

### Organ index

Each broiler’s heart, liver, and kidneys were harvested, and their weights were recorded. The organ weight (mg) was divided by the pre-slaughter broiler weight (g) to produce the organ index (mg/g), according to the following formula: organ index = (organ weight/broiler weight) × 100。.

### Histological analysis

The harvested small intestine samples were fixed in 4% % PFA solution for 24–48 h, dehydrated using an ascending concentration series of alcohol, paraffin-embedded, and sectioned to 5 μm thickness before staining with hematoxylin and eosin. In each section, ten whole crypt-villus units were selected in a random manner (one section per broiler, two broilers/replicate, five replicates/treatment). The distance measured vertically from the villus tip or crypt base to the villus-crypt junction was used to determine villus height (VH) and crypt depth (CD), correspondingly [15]. The histologic sections were examined with an Eclipse Ci-L microscope (Nikon, Japan). VH and CD were determined using Image-Pro Plus 6.0 (Media Cybemetics, U.S.A) after which the VH/CD ratio was computed.

### Serum biochemistry and antioxidant capacity

Using an automated biochemical analyzer (Cobas 8000, Roche Diagnostics, Switzerland), we determined the serum content of low-density lipoprotein (LDL), total cholesterol (TC), albumin (ALB), triglyceride (TG), alanine aminotransferase (ALT), aspartate aminotransferase (AST), high-density lipoprotein (HDL), and total protein (TP).

Serum levels of IgG, IgA, tumor necrosis factor α (TNF-α), IgM, interleukin-2 (IL-2), IL-4, and IL-8 were assessed through enzyme-linked immunoassays (ELISA) (double antibody sandwich method).

Serum levels of malondialdehyde (MDA) and glutathione peroxidase (GSH-Px) activities, superoxide dismutase (SOD) activities, and total antioxidant capacity (T-AOC) were determined using respective commercial test kits (MDA, A003-1; GSH-Px, A005, SOD, A001-1; T-AOC, A015-2-1; Nanjing Jiancheng Bioengineering Institute, Nanjing, Jiangsu, China) following the manufacturer’s instructions.

### Statistical analysis

Statistical analyses were executed using SPSS 22.0 (SPSS Inc., Chicago, IL, USA) utilizing one-way ANOVA and Duncan’s multiple range test for multiple comparisons. Duncan’s multiple comparison technique was employed to compare the means and test differences between treatments. Statistical significance is reported at *P* < 0.05.

## Results

### Components determination

**Fig. 1:**
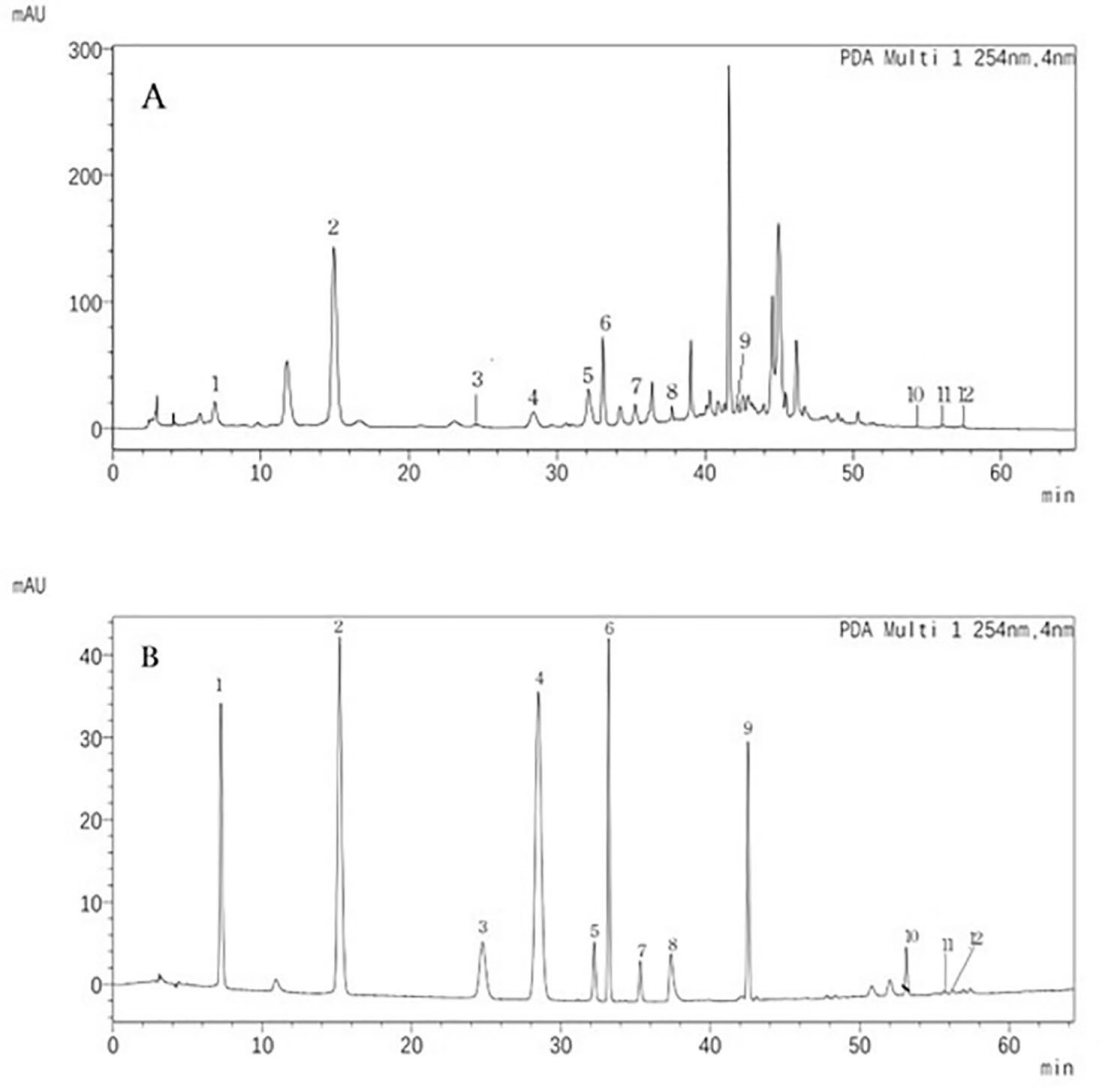
Illustrative HPLC chromatograms of (**A**) and mixed standards, (**B**). The compound numbers correspond to those listed in Table 2

Due to the complexity of the chemical constitution of ICFE, the twelve investigated components in ICFE were determined by the external standard method (Table 2). Figure 1 depicts the ICFE chromatographic profile produced under the aforementioned parameters.

**Table 2:** HPLC analysis of the 12 marker compounds

No.	Compounds	Retention time (min)	Contents (%)	
1	Gallic acid	7.22	0.21	
2	Protocatechuic acid	15.17	0.88	
3	Protocatechuic aldehyde	24.74	0.17	
4	P-hydroxybenzoic acid	28.48	0.54	
5	Chlorogenic acid	32.22	0.60	
6	Syringin	33.18	0.48	
7	Caffeic acid	35.30	0.10	
8	Chicoric acid	37.36	0.04	
9	Ferulic acid	42.51	0.05	
10	Caffeic acid ethyl ester	53.11	0.02	
11	Pedunculoside	55.67	1.52	
12	Caftaric acid	56.23	0.12	

### Growth performance

The effects of dietary ICFE supplementation on the growth performance of broilers at various ages are shown in Table 3. ICFE supplementation markedly increased the ABW at 22 and 42 days. Broilers receiving 500 and 1,000 mg/kg ICFE showed a substantially higher ADFI than the controls in the grower stage and overall (*P* < 0.05). Broilers receiving 250,500, and 1,000 mg/kg ICFE showed substantially higher ADG in the starter stage and overall (*P* < 0.05), compared to that of the controls. No significant effects on the FCR were observed in any treatment. Thus, broilers receiving 500 or 1,000 mg/kg ICFE exhibited better feed efficiency.

**Table 3:** Effects of ICFE supplementation on growth performance of broiler chickens^1^

Parameter	ICFE (mg/kg)						
	0	250	500	1000	SEM	*P*-Value	
ABW(g)							
**1 d**	32.88	33.63	33.00	33.25	0.18	0.47	
**22 d**	916.25^c^	942.88^b^	954.25^a^	950.13^a^	2.89	0.01	
**42 d**	2112.25^b^	2200.25^a^	2272.13^a^	2227.38^a^	17.34	0.01	
**ADFI(g)**							
**Starter (1–21 d)**	50.751^b^	52.05^a^	51.88^ab^	52.42^a^	0.22	0.04	
**Grower (22–42 d)**	117.76^c^	120.45^bc^	125.94^a^	123.20^ab^	0.90	0.01	
**Overall (1–42 d)**	84.24^c^	86.25^bc^	88.92^a^	87.82^ab^	0.49	0.01	
**ADG(g)**							
**Starter (1–21 d)**	41.49^c^	42.88^b^	43.39^a^	43.25^ab^	0.15	0.01	
**Grower (22–42 d)**	56.95^b^	59.88^ab^	62.76^a^	60.82^ab^	0.74	0.04	
**Overall (1–42 d)**	49.26^b^	51.36^a^	53.07^a^	52.01^a^	0.41	0.01	
**FCR((g/g)**							
**Starter (1–21 d)**	1.22	1.21	1.20	1.21	0.01	0.21	
**Grower (22–42 d)**	2.07	2.01	2.01	2.03	0.01	0.45	
**Overall (1–42 d)**	1.71	1.68	1.68	1.69	0.01	0.27	

^1^Data are means collected from 2 broiler/replicate, 9 replicates/treatment

^2^Abbreviations: ABW, average body weight; ADFI, average daily feed intake; ADG, average daily gain; FCR, feed conversion rate. ^3^ICFE, Ilicis chinensis folium extract; ^4^SEM, total standard error of means. ^5 a,b,c^ Means with different letters within a row differ significantly (*P* < 0.05)

### Organ index

The effects of ICFE supplementation on the organ indices are shown in Table 4. No significant differences in organ indices were observed between the experimental and control groups (*P* > 0.05).

**Table 4:** Effects of dietary ICFE supplementation on the organ index of broiler chickens^1^

Parameter	ICFE (mg/kg)				SEM	*P*-Value
	0	250	500	1000		
**heart**	0.45	0.42	0.46	0.43	0.01	0.98
**liver**	2.15	2.21	2.10	2.20	0.05	0.21
**spleen**	0.11	0.10	0.11	0.11	0.01	0.91

^1^Data are means collected from 2 broiler/replicate, 9 replicates/treatment

### Intestinal morphology

Broilers receiving 250 and 500 mg/kg ICFE supplementation showed greater VH in the duodenum compared with that of the controls, and this difference was more pronounced (*P* < 0.05, Table 5) in the 1000 mg/kg ICFE treatment. ICFE-supplemented birds produced substantially higher duodenum VH/CD values (*P* < 0.05). CD was generally lower in the ICFE-supplemented broilers than in the controls (*P* < 0.05), and VH in the jejunum was greater in the ICFE treatments than in the controls but not statistically significant. In the ileum, supplementation with 250 mg/kg ICFE significantly increased VH and reduced CD, compared to the controls.

**Table 5:** Effect of dietary different concentration of ICFE supplementation on intestinal morphology of broiler chickens^1^

Parameter	ICFE (mg/kg)							
	0	250	500	1000	SEM		*P*-Value	
Duodenum								
**VM, mm**	1.29^b^	1.50^a^	1.62^a^	1.28^b^	0.04		0.01	
**CD, mm**	0.26^a^	0.24^ab^	0.24^ab^	0.18^b^	0.01		0.03	
**VH/CD**	4.99^b^	6.61^a^	6.87^a^	7.22^a^	0.31		0.04	
**jejunum**								
**VM, mm**	1.27	1.31	1.29	1.27	0.01		0.45	
**CD, mm**	0.17^b^	0.23^a^	0.22^a^	0.19^b^	0.01		0.01	
**VH/CD**	7.35^a^	5.76^b^	5.89^b^	6.75^ab^	0.23		0.03	
**ileum**								
**VM, mm**	1.06^b^	1.19^a^	0.84^d^	0.98^c^	0.03		0.01	
**CD, mm**	0.23^bc^	0.27^a^	0.26^ab^	0.22^c^	0.01		0.01	
**VH/CD**	4.58^a^	4.49^a^	3.26^b^	4.76^a^	0.20		0.01	

^1^Data are means collected from 2 broiler/replicate, 9 replicates/treatment

^2^Abbreviations: VH, villus height; CD, crypt depth; VH/CD, villus height/crypt depth. ^a,b,c^ Means with different letters within a row differ significantly (*P* < 0.05)

### Serum biochemistry

The ALT, AST, TP, ALB, and LDL levels were generally lower in the ICFE treatments than in the controls (Table 6), however, no significant effect of any dosage was observed.

**Table 6:** Effect of dietary different concentration of ICFE supplementation on serum biochemistry of broiler chickens^1^

Parameter	ICFE (mg/kg)				SEM	*P*-Value
	0	250	500	1000		
**ALT(U/L)**	1.14	1.02	1.09	0.88	1.10	0.83
**AST(U/L)**	402.12	304.20	321.67	332.25	19.64	0.32
**TP(g/L)**	51.10^a^	43.40^ab^	36.00^b^	41.50^ab^	2.15	0.04
**ALB(g/L)**	17.00	13.80	12.80	14.30	0.90	0.40
**LDL(mmol/L)**	1.112	0.85	0.79	0.95	0.07	0.39
**HDL(mmol/L)**	1.81^ab^	1.76^ab^	1.67^b^	2.14^a^	0.07	0.02
**TC(mmol/L)**	3.05	2.77	2.77	3.01	0.11	0.24
**TG(mmol/L)**	0.72^ab^	0.51^b^	0.94^a^	0.62^ab^	0.07	0.01

^1^Data are means collected from 2 broiler/replicate, 9 replicates/treatment

^2^Abbreviations: ALT, alanine aminotransferase; AST, aspartate aminotransferase; TP, total protein; ALB, Albumin; HDL, high-density lipoprotein cholesterol; LDL, low-density lipoprotein cholesterol; TC, total cholesterol; TG, triglyceride

^3 a−b^Means with different letters within a row differ significantly (*P* < 0.05)

### Antibody and cytokine levels in serum

Effects of ICFE supplementation on antibody and cytokine concentrations in the serum of broilers are presented in Table 7. Supplementation with ICFE increased the serum IL-2, IL-4, IL-8, and TNF-α levels; in particular, broilers receiving a 1,000 mg/kg ICFE showed higher IL-2 and IL-4 levels than the controls. The serum IgA levels of broiler chickens receiving 250 mg/kg ICFE were significantly higher than those of the control (*P*<0.05). None of the ICFE treatments significantly affected the IL-8, TNF-α, IgG, and IgM levels in the serum.

**Table 7:** Effect of dietary different concentration of ICFE supplementation on antibodies and cytokines in serum of broiler chickens^1^

Parameter	ICFE (mg/kg)				SEM	*P*-Value
	0	250	500	1000		
**IL-2 (ng/L)**	8.62^b^	18.24^ab^	12.01^ab^	38.524^a^	4.62	0.01
**IL-4 (ng/L)**	53.57^b^	105.02^ab^	84.45^ab^	115.62^a^	10.52	0.17
**IL-8 (ng/L)**	566.01	663.48	626.67	751.97	46.97	0.58
**TNF-α (ng/L)**	39.01	74.13	55.81	83.46	8.51	0.26
**IgG (mg/ml)**	18.41	17.89	17.25	20.77	1.59	0.88
**IgA (mg/ml)**	3.09	6.19^a^	4.51^b^	4.44^b^	0.32	0.01
**IgM (mg/ml)**	21.18	20.50	26.09	26.99	2.27	0.66

^1^Data are means collected from 2 broiler/replicate, 9 replicates/treatment

^2^Abbreviations: IL-2, Interleukin-2; IL-4, Interleukin-4; IL-8, Interleukin-8; TNF-α, tumor necrosis factor-α; IgG, immunoglobulin G; IgA, immunoglobulin A; IgM, immunoglobulin M

^3a−b^Means with different letters within a row differ significantly (*P* < 0.05)

### Antioxidant capacity

The effects ICFE supplementation on the activities of SOD, GSH-Px, and T-AOC, and the MDA levels in the serum of broilers at 42 days of age are shown in Table 8. The activities of SOD and T-AOC in the serum were elevated in boilers receiving ICFE supplement (*P* > 0.05, each). ICFE at 500 and 1,000 mg/kg reduced the serum MDA levels, and GSH-Px activities were reduced in all ICFE supplementation treatments.

**Table 8:** Effects of dietary ICFE supplementation on superoxide dismutase (SOD), glutathione peroxidase(GSH-Px) and total antioxidant capacity (T-AOC) activities and malondialdehyde(MDA) concentration in serum of broiler chickens at 42D of age^1^

Parameter	ICFE (mg/kg)				SEM	*P*-Value
	0	250	500	1000		
**SOD (U/mL)**	717.94^b^	875.96^a^	917.63^a^	861.75^ab^	27.24	0.05
**GSH-Px(U/mL)**	684.94	653.67	533.24	659.78	54.94	0.78
**MDA (nmol/mL)**	5.98	6.39	5.42	5.55	0.37	0.79
**T-AOC (mM)**	0.77	0.82	0.81	0.84	0.02	0.75

^1^Data are means collected from 2 broiler/replicate, 9 replicates/treatment

## Discussion

Broiler chickens and pigs may benefit from diets supplemented with Chinese herbal concoctions such as *Amaranthus hybridus*, *Astragalus, Glycyrrhiza* and *Camellia sinensis* which enhance growth performance, strengthen the immune system, increase antioxidant activity, and help prevent infections [16–18].

Reverse-phase HPLC coupled to a diode array detector (RP-HPLC-DAD) was applied to characterize the components of ICFE. The following compounds were observed: ferulic acid, chlorogenic acid, caffeic acid, caffeic acid ethyl ester, chicoric acid, gallic acid, protocatechuic acid, p-hydroxybenzoic acid, protocatechuic aldehyde, caftaric acid, pedunculoside, and syringin. pedunculoside (Fig. 1). The content of pedunculoside was the highest, and that of protocatechuic acid ranked second (Table 2).

The effects of ICFE supplementation on the growth performance of broilers have never been investigated, to our knowledge. Our results showed that supplementation with ICFE increased ABW, ADFI, and ADG through elevated VH and VH/CD values in the jejunum and duodenum and helped digestive and absorption processes, which resulted in better intestinal health. Increased villi height facilitates better small intestinal absorption, and reduced digestion and absorption ability is associated with increasing crypt depth, and when the VH/CD is high, digestion and absorption are enhanced, suggesting that the ability to digest and absorb nutrients in the gut is favorably linked to the VH/CD ratio [19, 20]. As discussed above, ICFE contains high levels of polyphenol antioxidants including chlorogenic, protocatechuic, gallic, caffeic, and chicoric acids. We propose that ICFE may improve the growth performance of broiler chicks considering that phenolic compounds were associated with the ability of ICFE to change the intestinal morphology, which improved the ability to absorb nutrients from their regular diet [21].

As expected, dietary supplementation of ICFE did not damage the heart, liver, and spleen in the present study. There were no gross pathological changes in the internal organs of the broiler chickens in each ICFE group relative to the controls which indicated low toxicity of adding ICFE for a long-term.

Dietary ICFE supplementation decreased the ALT, AST, LDL, TP, TC and ALB levels of broilers in the current study, compared to those of the controls. *Ilex paraguariensis* can exert substantial hypolipidemic effects, lowering the serum levels of TG and LDC-cholesterol (LDL-C), and TC increases as a result of a high-cholesterol diet [22], which is in line with our findings that dietary supplementation with ICFE may lower TC and LDL content; however, the effect was not significant. Pedunculoside can lower TC and LDL-C and reduce hepatic TC in high-fat diet-induced hyperlipidemia rats [23]. Triterpenoid glycoside with anti-inflammatory [24], anti-arrhythmia, and lipid-lowering effects such as pedunculoside and syringin is a further typical compound of ICFE [23]. Additionally, pedunculoside markedly attenuates the synthesis of COX-2, IL-6, TNF-α, IL-1β, and Inos in RAW264.7 macrophages during LPS-mediated inflammatory responses [25]. Thus, the reduction in serum levels of ALT, LDL, and TC through ICFE supplementation confirmed the beneficial effects on broiler health.

Serum immunoglobulins include key markers of the immunological state of an animal [26, 27]. A previous study showed that ethanol extracts from *Ilex pubescens* can downregulate the expression levels of inflammatory cytokines and substances including COX-2, IL-6, MIP-1α, IL-1β, MIP-1, and TNF-α while increasing the abundances of anti-inflammatory cytokines such as IL-10 and TGF-β [28]. Proinflammatory cytokines are produced predominantly by activated macrophages and are involved in the upregulation of inflammatory reactions [29]. Consistent with these facts, we observed in the present study that dietary ICFE supplementation promoted the humoral immune response of broilers, leading to an increase in the abundances of IL-4 and IgA in the serum owing to the stimulation of immune responses.

The results of the current study indicated that ICFE can promote antioxidant activity according to the levels of various antioxidant markers in the serum, such as SOD and T-AOC. To exert their role as antioxidants, SOD and T-AOC may scavenge newly generated reactive oxygen species (ROS). SOD is responsible for the initial breakdown of superoxide into hydrogen peroxide, after which an enzyme cascade including GSH-Px completes the final conversion to water [30]. MDA is a common metric for measuring lipid peroxidation. Damage to cell membranes, which leads to an increase in MDA, is the first effect of a surge in ROS production [31]. In this current study, supplementing broiler diets with ICFE boosted SOD and T-AOC functions and lowered MDA concentrations in the serum. Our phytochemical analysis of ICFE revealed a high abundance of phenolics and triterpenoid glycosides. Similarly, plant extracts rich in flavonoids, phenolic chemicals, and triterpenoids have also been demonstrated to exhibit a strong association with antioxidant activity in multiple in vitro studies [32–35]. For example, phenolic compounds such as caffeic acid, chlorogenic acid and rutin are the primary components in *Ilex paraguariensis* which are associated with antioxidant functions [36]. Elevated levels of polyphenols such as chlorogenic acid, p-hydroxybenzoic acid, protocatechuic acid, gallic acid, and caftaric acid were also found in ICFE through RP-HPLC-DAD analysis. Thus, we propose that the antioxidant effects of ICFE supplementation may be due to its phenolic and triterpenoid compounds, which may increase ABW, ADFI, ADG and improve the growth performance of broiler chickens [37].

## Conclusion

We evaluated the potential of ICFE as an alternative to in-feed antibiotics and demonstrated that supplementing broiler chicken diets with ICFE may remarkably enhance overall period ABW and ADG. To our knowledge, this study is the first to show that ICFE is a beneficial and effective feed supplement for enhancing the growth performance of broiler chickens. Our findings provide a better understanding of the mechanism of growth-promoting effects of ICFE supplementation. We recommend ICFE supplementation in diets of broiler chicks at 500 mg/kg because of its unique properties and positive effects on poultry production.

## Data Availability

All data generated or analyzed during this study are available from the corresponding author upon reasonable request.

## Abbreviations

ABW: Average body weight
ADFI: Average daily feed intake
ADG: Average daily gain
FCR: Feed conversion rate
ICFE: Ilicis chinensis folium extract
SEM: Total standard error of means
VH: Villus height
CD: Crypt depth
VH/CD: Villus height/crypt depth
ALT: Alanine aminotransferase
AST: Aspartate aminotransferase
TP: Total protein
ALB: Albumin
HDL: High-density lipoprotein cholesterol
LDL: Low-density lipoprotein cholesterol
TC: Total cholesterol
TG: Triglyceride
IL-2: Interleukin-2
IL-4: Interleukin-4
IL-8: Interleukin-8
TNF-α: Tumor necrosis factor-α
IgG: Immunoglobulin G
IgA: Immunoglobulin A
IgM: Immunoglobulin M
SOD: Superoxide dismutase
GSH-Px: Glutathione peroxidase
T-AOC: Total antioxidant capacity
MDA: Malondialdehyde
